# Endovascular Occlusion of a Renal Arteriovenous Fistula with Renal Vein Aneurysm Formation for Rupture Prevention

**DOI:** 10.1155/2019/8530641

**Published:** 2019-10-31

**Authors:** Yae Hyun Rhee, Lucas Busch, Roberto Sansone, Neslihan Ertas, Nikolaos Floros, Hubert Schelzig, Joscha Mulorz, Markus Udo Wagenhäuser

**Affiliations:** ^1^Department of Vascular and Endovascular Surgery, Medical Faculty, Heinrich-Heine-University Düsseldorf, Moorenstraße 5, 40225 Düsseldorf, Germany; ^2^Cardiovascular Research Institute Düsseldorf (CARID), Division of Cardiology, Pulmonology, and Vascular Medicine, University Duesseldorf, Medical Faculty, Moorenstraße 5, 40225 Düsseldorf, Germany

## Abstract

**Purpose:**

To report the effectiveness of left renal artery (LRA) occlusion using Amplatzer Vascular Plug (AVP) II as treatment for a high-flow renal arteriovenous fistula (RAVF) with multiple renal vein aneurysms (RVA) to prevent aneurysm rupture and cardiac decompensation.

**Case Report:**

A 59-year-old female suffering from a post-traumatic RAVF presented with tachycardia and increased cardiac output (CO). Doppler ultrasonography and computed tomography (CT) scan revealed a high-flow RAVF with multiple RVAs and unilateral critically reduced kidney function. Appreciating recent interventional therapeutic advances, the patient was treated with endovascular placement of AVP II into the left renal artery (LRA) resulting in complete occlusion of the RAVF to effectively reduce the risk of RVA rupture and cardiac decompensation. No anti-platelet medication was administrated after the occlusion of the LRA. The patient's physical capacity improved since right heart volume strain was normalized, and CO was reduced.

**Conclusion:**

Transbrachial AVP II occlusion of the LRA is effective to occlude high-flow RAVFs to prevent risk of life-threatening RVA rupture. Additional follow-up is warranted to verify long-term effectiveness of this approach.

## 1. Introduction

Renal arteriovenous fistula (RAVF) is a rare entity with a rate of incidence less than 0.04% on post-mortem histopathological examinations [1]. The most common symptoms of RAVF include abdominal bruit, gross or micro-haematuria, diastolic arterial hypertension and increased cardiac output. Ultimately, the decreased peripheral resistance may lead to diastolic overload and congestive cardiac failure [2, 3]. Remarkably, acquired RAVF can display aneurysmal appearance and is considered to bear high risk for fatal rupture with life-threatening bleeding complications [4].

Primary renal vein aneurysms (RVA) have a low prevalence with only few cases published [5–8]. Vein aneurysms in general are characterized by a thin aneurysm wall due to marked medial atrophy and elastic fibre loss [9]. Interestingly, simultaneous occurrence of an acquired RAVF and RVA has been described in rare cases [10, 11].

Here, we report the case of a 59-year-old woman with a high-flow RAVF combined with multiple RVAs. This was successfully treated with the Amplatzer Vascular Plug (AVP) II device (Abbott Vascular, Abbott Park, IL, USA) for complete RAVF occlusion and prevention of RVA rupture.

## 2. Case Presentation

A 59-year-old female was admitted to our Department of Vascular and Endovascular Surgery from her general practitioner who suspected an RAVF in Doppler ultrasonography.

The patient reported suffering from mild shortness of breath during normal everyday activities (NYHA II). The patient did not suffer from any relevant co-morbidity or allergies and had no former pregnancies or previous surgeries. Notably, the patient reported a blunt chest trauma from physical contact during a karate class more than 30 years ago. At that time, X-ray examination revealed multiple rib-fractures in the left upper thorax which were treated conservatively.

**Figure 1:**
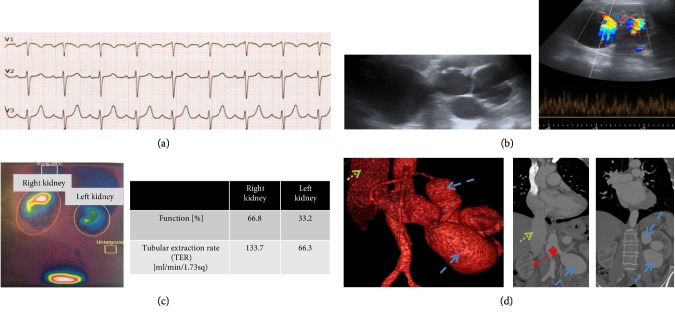
Clinical finding and imaging prior to renal arterio-venous fistula (RAVF) occlusion. (a) Electrocardiogram (ECG) with sinus tachycardia in precordial right heart leads V1–3. No pathological ECG intervals. (b) multiple renal vein aneurysm (RVA) with broadening of spectral waveforms suggesting arterial aneurysm perfusion. (c) Renal scintigraphy (MAG-3) found a split renal function of left: 33% vs. right: 67% since a proximal high-flow RAVF caused reduced kidney perfusion. (d) 3D and coronal reconstruction. RVA (blue arrows, dashed) which are likely to be a long-term sequalae from a proximal high-flow RAVF. Recognize the straight proximal segment of the left renal artery (LRA) (red arrow, dashed, and dotted) for Amplatzer Vascular Plug (AVP) placement. Recognize the diameter enlargement of the Inferior Vena Cava (IVC) (green arrow, dotted) caused by increased volume load.

On physical examination the patient was normotensive while the electrocardiogram (ECG) revealed a resting sinus tachycardia (Figure 1(a)). While obvious signs of right heart strain such as murmur, accentuated second heart sound, diaphoresis, lower extremity edema or cyanosis were absent, we observed elevated jugular vein pulse (JVP) indicating significant venous hypertension (Supplementary [Supplementary-material supplementary-material-1]). The abdomen was soft during deep palpation with no abdominal masses detected. Intestinal peristalsis was normal. Interestingly, we found a palpable thrill and an auscultable bruit at the patient's left flank. The assessment of peripheral pulses revealed normal intensity rate with no palpable tenderness at any location.

Abdominal Doppler ultrasonography revealed multiple RVAs and the typical broadening of spectral waveform suggested a RAVF with arterial flow (Figure 1(b)). Renal scintigraphy revealed split residual renal function of 33% vs. 67% (left vs. right kidney) (Figure 1(c)). Contrast-enhanced computed tomography (CT) findings suggested a RAVF along with an enlargement in the diameter of the LRA, left renal vein (LRV) and multiple RVAs at a maximum diameter of 5.5 × 7.2 cm. Notably, the high-flow RAVF also caused an enlargement of the maximum diameter of the Inferior Vena Cava (IVC) (41 mm). Further, compression of the left ureter and subsequent urinary retention was diagnosed. The diameter-enlarged LRA mimicked the aortic bifurcation with a similar take-off angle and a straight proximal segment (Figure 1(d)).

**Figure 2:**
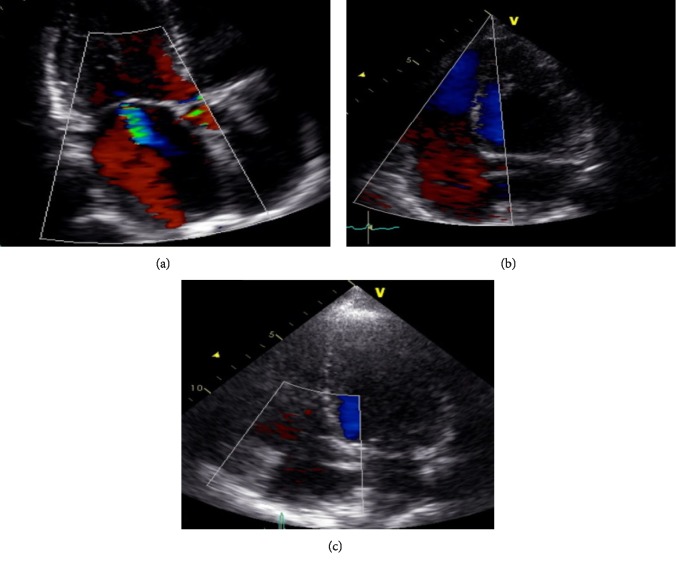
Echocardiography before (a), 2-days (b), and 3-months (c) after occlusion of the proximal high-flow renal arteriovenous fistula (RAVF) by Amplatzer Vascular Plug (AVP) Device II placement into the left renal artery (LRA). (a) 4-Chamber view with Doppler ultrasound found a functional (secondary) tricuspid regurgitation (Grade I). Recognize the enlargement of the right atrium due to increased volume load. (b and c) 4-Chamber view with Doppler ultrasound found no tricuspid regurgitation 2-days and 3 months after RAVF occlusion. Also recognize normalization of the right atrium dimensions due to reduced volume load.

The pre-interventional echocardiography demonstrated signs of substantial right heart strain with functional tricuspid regurgitation and right atrium enlargement due to increased volume load (Figure 2(a)).

Taking the above into consideration, there was interdisciplinary consultation with the radiology department. Following a rigorous risk-benefit assessment of different therapeutic approaches, an endovascular occlusion of the LRA to treat the RAVF was preferred.

After informed consent was obtained, we performed the complete endovascular occlusion of the LRA via transbrachial access route since the risk for cerebral events was considered acceptable in absence of atherosclerotic lesions. To measure the target vessel, the authors utilized the open-source imaging software OsiriX, version 7.5 for AVP deployment.

Surgery was performed in a hybrid operation room, considering the moderate risk of accidental aneurysm rupture during guide wire advancing and AVP II placement.

**Figure 3:**
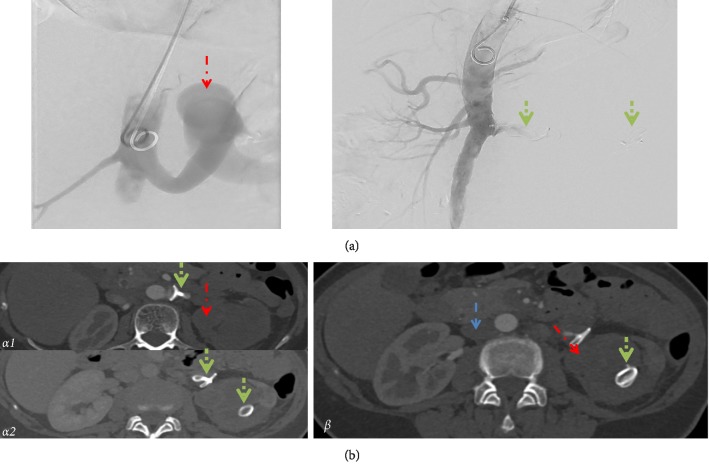
Representative images of an angiography during surgery and a CT scan one-month post-surgery. (a) Angiography before and after Amplatzer Vascular Plug (AVP) II Device placement. No perfusion of the left renal artery (LRA) after AVP II placement. Red arrow (dashed and dotted) = RVA; Green arrow (dotted) = correct positioned central and displaced peripheral AVP II. (b) *α*: arterial phase images. *α*1: no perfusion of the LRA distal of the central AVP II. *α*2: minimal residual cortical perfusion over a side-branch originating from the LRA proximal of the central AVP II. *β*: venous phase image: no contrast in the left vs. right kidney indicating subtotal infarction, despite insignificant residual cortical perfusion. No residual perfusion of the RVA (red arrows, dashed, and dotted) and marked reduction of the IVC diameter (blue arrow, dashed).

In short, after advancing a pigtail catheter into the aorta the LRA was marked using aortography. The LRA was cannulated with a right-modified catheter after carefully advancing a rosen wire. Following correct positioning, the first AVP II device (20 mm) was released, however, control angiography showed secondary dispositioning as the device travelled with the blood stream into the distal part of the LRA. Since the authors considered maldeployment being the most likely reason for secondary mispositioning, a second AVP II device of the same dimension was placed correctly into the proximal part of the LRA close to its origin from the aorta. The subsequent control angiography confirmed the complete occlusion of the LRA with no residual perfusion, demonstrating successful blocking of arterial perfusion of the RVA (Figure 3(a)).

Absence of blood flow in the LRA and in the left kidney was confirmed two days after intervention with Doppler sonography. Maximum elevation of serum LDH levels (peak: 900 U/l) corroborated the ultrasound findings.

A three-day post-intervention echocardiography found a reduced cardiac volume load along with a normalization of the cardiac output and heart rate (Supplementary [Supplementary-material supplementary-material-1]). In comparison to pre-intervention findings, there was no residual tricuspid regurgitation and the right atrium dimensions were reduced (Table 1, Figures 2(b) and 2(c)). No procedural or post-interventional complications occurred. The patient was discharged 7-days post-intervention in stable condition.

A follow-up CT scan after one month revealed appropriate positioning of the proximal AVP II without residual side perfusion. Notably, a small proximal side-branch sustained an insignificant residual cortical renal perfusion (Figure 3(b)). Further, the CT scan confirmed subtotal infarction of the left kidney and marked reduction of IVC diameter (Figure 3(b)). On clinical examination, the patient reported a significantly improved physical capacity (NYHA I). Serum creatinine levels were normal suggesting sufficient compensation by the right kidney with sustained normal diuresis volume.

On three-month follow-up examination the patient presented in a good general condition and reported arterial hypertension, for which she was treated with amlodipine. The Doppler ultrasound detected no residual perfusion of the left kidney and revealed a modest size reduction. Further, a second follow-up echocardiography revealed normal right atrial dimensions as well as normalization of cardiac strain parameters (Supplementary [Supplementary-material supplementary-material-1]).

## 3. Discussion

We presented the rare case of a 59-year old female suffering from a high-flow RAVF of uncertain aetiology causing multiple RVAs at high risk of rupture, successfully treated by endovascular occlusion of the LRA utilizing AVP II.

Based on their aetiology, RAVF have been classified into congenital (14–27%), acquired (70–80%) and idiopathic (3%) sub-types [12]. These sub-types differ in their angiographic appearance. In detail, congenital RAVFs have a cirsoid, knotted structure with multiple interconnecting fistula between the feeding artery and draining vein. Different to congenital RAVFs, idiopathic and acquired types only consist of single arteriovenous interconnection. Erosion of arterial wall into the adjacent vein leading to the fusion of the vessel walls is considered the underlying pathophysiological mechanism [13, 14]. Acquired RAVF is the most common sub-type and is considered a sequel of iatrogenic, blunt or even penetrating renal trauma [15–20]. Less frequent pathologies causing RAVF development include malignancy with tumor neovascularity [14] and/or focal inflammation of the vessel wall [2]. The interval between the initiating trauma and diagnosis of RAVF varies from a couple of weeks to several years [16, 20]. Spontaneous regress has been reported for small numbers of posttraumatic AVFs [17]. Given that current literature report cases of delayed RAVF development after blunt chest trauma, it makes a causal association for our case likely [21]. However, as the exact time point of RAVF development in our patient remains uncertain, the exact aetiology cannot be ultimately determined.

As of today, little is known about the pathogenesis of RVA due to low prevalence. In general, congenital RVA may arise from embryologic mis-development due to the complex process of vein formation [22]. Aside from this theory, there may be other potential morbidities causing RVA development, such as IVC thrombosis, RAVFs and chronic renal vein hypertension of the left renal vein due to permanent compression between the superior mesenteric artery and aorta-also known as nutcracker phenomenon [6, 23].

In particular, Martinez et al. reported the association between RAVF and RVA, suggesting long-term high-flow and -pressure strain of the renal vein to cause secondary dilatation with ultimate RVA development [24]. In such a scenario, medial vein wall atrophy is the histological equivalent leading to pathological wall weakening, ultimately translating into RVA development [9].

For the case described above, several aspects had to be considered for an adequate risk-benefit assessment to suggest the patient the most beneficial treatment option. Taking this into consideration, there is a general agreement that the diameter of RVAs is associated with rupture risk [11]. Next, the thickness of the aneurysm wall, which is reduced in venous compared to arterial aneurysms, also critically contributes to RVA rupture [4]. Lastly, the impaired quality of life as a result of reduced physical capacity due to substantial bypass volume through the RAVF must be considered. Based on these considerations, we suggested urgent therapy for our patient.

Until the end of the last millennium, open total or partial surgical nephrectomy was considered as standard therapy to treat symptomatic RAVF [16]. Although partial surgical nephrectomy may preserve renal function, this approach bears a high risk of intraoperative RVA rupture and subsequent bleeding complications during the surgical side access that were potentially life-threatening. Considering the dimensions of the RVAs and close proximity to the abdominal wall and an inaccessible clamping zone in case of unintentional RVA rupture during surgery, we decided, after rigorous risk-benefit assessment, for an endovascular approach prioritizing the patient's safety. Nevertheless, the authors are aware that the so chosen endovascular treatment accepts an intentional total renal infarction which in turn, may increase the risk for subsequent infection.

To maintain the patient's bilateral kidney function, renal auto transplantation was taken into consideration. In fact, there are several indications for renal auto transplantation described in the current literature, including ureteral strictures and complex vascular abnormalities affecting renal arteries [25]. The procedure had been first described by James Hardy in 1963, who treated complex ureteral injuries during aortic surgery. Considering the recent advances of endovascular techniques, renal auto transplantation for treatment of vascular abnormalities became less common during the last decades [26]. For the case presented above, we decided against this invasive approach, since we considered an imminent risk of RVA rupture during open surgery.

For the case presented above, we considered several potential endovascular therapy options. However, the implantation of a covered stent to occlude the RAVF did not seem promising for the presented case since any pre-surgery imaging could not detect the exact localization of the RAVF.

The implantation of a fenestrated aortic stentgraft covering the LRA and blocking the RAVF perfusion may seem feasible to reduce the rupture risk of the multiple RVAs.

Following the same concept, renal artery embolization is an endoluminal therapy based on partial or super selective occlusion through a minimal invasive access route [27]. Several occluding devices and materials are available, such as coils with or without wall stents, cyanoacrylate glue, covered stents, detachable balloons, gelfoam sponge and liquids [28–31]. Although this approach may potentially preserve kidney function, it is technically demanding.

In 1973, Bookstein and Goldstein first introduced selective renal artery embolization to control haemorrhage after a stab wound [32, 33]. Ever since, endovascular approaches have led to a drastic decrease in mortality, morbidity and hospital time when compared to conventional surgery and thus have replaced open surgery as gold standard therapy for various indications [34].

AVP II is one of the most recent developed devices and promises full cross-sectional vessel coverage accepting complete end organ infarction. The first placement of such AVP II was reported by Tabori and Love in pulmonary AVF, while Perkov et al. reported successful embolization of high-flow RAVF in 2012 [34, 35]. This device is known for its flexibility and its precise (re)deployment capability [36, 37]. While multiple coils are needed to embolize an RAVF, only a single AVP II is necessary to achieve complete occlusion of the vascular connection [38, 39]. Notably, reported spontaneous recanalization rates after occlusion are low [36]. The AVP II is self-expanding and provides sufficient radial force to the vessel wall, minimizing micromovements and plug migration at long-term. According to the manufacturer's recommendation, oversizing of 30–50% of the maximum target vessel diameter is suggested to increase the stability [36]. In addition to these advantageous properties, the risk of coil migration to the heart and pulmonary artery due to the high-flow of an RAVF can be reduced by utilizing the AVP II [40, 41]. All the aspects mentioned above along with a pre-interventional unilateral reduced kidney function advocated using the AVP II device for the occlusion of the feeding LRA and the RAVF.

Since follow-up Doppler ultrasonography and CT-scan confirmed successful occlusion of the LRA with no residual RVA perfusion and reduced IVC diameter, it was decided not to occlude the LRV, assuming an insignificant residual rupture risk.

Renovascular hypertension is an established sequel of renal artery occlusion based on the Goldblatt-mechanism. Since our patient reported the onset of hypertension during the 3-month follow up examination, rigorous blood pressure control by a general practitioner is imperative.

We did not administrate any anti-platelet medication or anticoagulation after AVP II placement in the LRA for RAVF occlusion. This decision was backed-up by most reports in current literature suggesting only low risk of secondary pulmonary embolism from thrombus formation in the former draining vein [31, 38, 42]. As we did not observe LRV thrombus formation at three-month post-intervention, the administration of anticoagulant even seems dispensable in cases of RVA formation.

## 4. Conclusion

We hereby present a case of a rare and complex vascular disease bearing a high risk of rupture. RAVF was successfully treated by transbrachial occlusion of the LRA utilizing AVP II to block perfusion of the RVA to minimize risk of rupture. We did not administrate anticoagulation or anti-platelet medication and did not observe thrombus formation in the former draining vein. Further, clinical follow-up is required to detect leakage of the AVP II and/or secondary migration which might require re-intervention and to adequately monitor the secondary hypertension and its sequelae.

## Figures and Tables

**Table 1:** Clinical and cardiac parameters before and after the occlusion of the left renal artery (LRA). Listed are relevant clinical haemodyamic and cardiac parameters before, 2-days and 3-months post-surgery with placement of two Amplatzer Vascular Plug (AVP) II devices in the LRA causing the occlusion of the renal arterio-venous fistula (RAVF).

Clinical stage	Before	After	FU after 3 months
NYHA^a^	II	I	I

*Haemodynamics*			
Heart rate (bpm^b^)	115	70	77
Cardiac output (l/min)	11.6	6	4.2

*Echocardiogram*			
LVEDD^c^ (mm)	52	45	44
PW-EDWT^d^ (mm)	11	13	8
IVS-EDWT^e^(mm)	10	13	11
LEDV^f^ (ml)	148	121	95
LESV^g^ (ml)	53	36	40
LAA^h^ (cm^2^)	31	24	18
LAV^i^ (ml)	122	92	59
LVEF^j^ Simpson (%)	64	71	58
TR^k^grade (I–III)	I	None	None
sPAP^l^ (mmHg)	35	20	18
TAPSE^m^ (mm)	35	24	23
RVEDD^n^ (mm)	34	28	27
RAA^o^ (cm^2^)	26	21	20
RAV^p^ (ml)	56	51	50
VCD^q^ (mm)	30	Collapsed	16

^a^: New York Heart Association stage. ^b^: Beats per minute. ^c^: Left ventricular end-diastolic diameter. ^d^: Posterior wall end-diastolic thickness. ^e^: Interventricular septal end-diastolic thickness. ^f^: Left ventricular end diastolic volume. ^g^: Left ventricular end systolic volume. ^h^: Left atrial area. ^i^: Left atrial volume. ^j^: Left ventricular ejection fraction. ^k^: Tricuspid regurgitation. ^l^: Systolic pulmonary artery pressure. ^m^: Tricuspid annular plane systolic excursion. n: right ventricular end-diastolic diameter. ^o^: Right atrial area. ^p^: Right atrial volume. ^q^: Vena cava diameter.

## Supplementary Materials

Supplementary 1 Supplementary Video 1: Elevated jugular vein pulse (JVP) indicating significant venous hypertension.

Supplementary 2 Supplementary Figure 1: Heart rate (bpm) and cardiac output (CO) over 3 time course of 3 months.
