# Supplementary material for: Endovascular Occlusion of a Renal Arteriovenous Fistula with Renal Vein Aneurysm Formation for Rupture Prevention
Source: Case Rep Vasc Med. 2019 Oct 31;2019:8530641. doi: 10.1155/2019/8530641 (PMC6930724; doi:10.1155/2019/8530641)
Supplement: Supplementary 2 — Supplementary Figure 1: Heart rate (bpm) and cardiac output (CO) over 3 time course of 3 months. [file 8530641.f2.pdf]

Supplement Figure 1

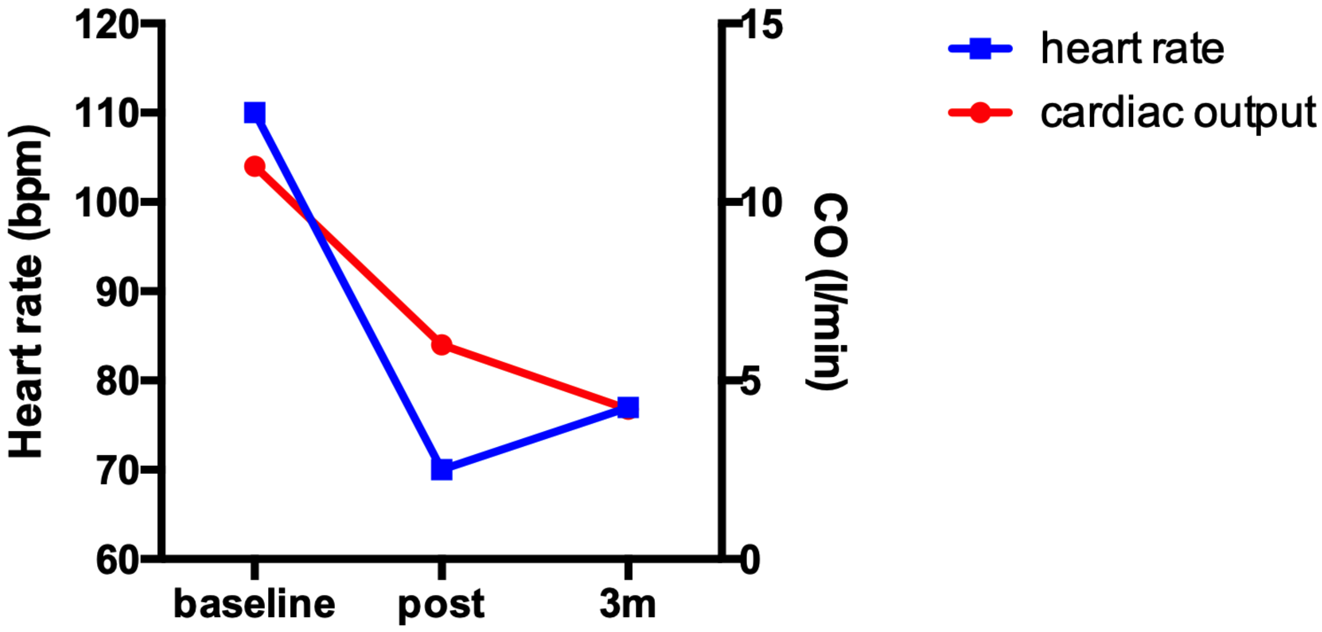

Supplement Figure 1: Heart rate (bpm) and cardiac output (CO) over 3 time course of 3 months. Elevated bpm and CO prior to treatment. Both parameters normalized two days (post) and 3 months (3m) after the occlusion of the RAVF utilizing Amplatzer Vascular Plug (AVP) Dev ice II. Concordantly, the patient's physical capacity improved.
